# Impact of different concurrent training sequencing schemes on overnight systemic immunological regulation in adolescent athletes

**DOI:** 10.3389/fphys.2025.1392946

**Published:** 2025-03-27

**Authors:** Thomas Steidten, Urs Granacher, Holger Gabriel, Simon Haunhorst, Philipp Reuken, Diana Dudziak, Christian Puta

**Affiliations:** ^1^ Department of Sports Medicine and Health Promotion, Friedrich-Schiller-University Jena, Jena, Germany; ^2^ Department of Sport and Sport Science, Exercise and Human Movement Science, University of Freiburg, Freiburg, Germany; ^3^ Center for Interdisciplinary Prevention of Diseases Related to Professional Activities, Friedrich-Schiller-University Jena, Jena, Germany; ^4^ Department for Internal Medicine IV (Gastroenterology, Hepatology and Infectious Diseases), Jena University Hospital, Jena, Germany; ^5^ Institute of Immunology, Jena University Hospital, Friedrich-Schiller-University Jena, Jena, Germany; ^6^ Center for Sepsis Control and Care (CSCC), Jena University Hospital/Friedrich-Schiller-University Jena, Jena, Germany

**Keywords:** leucocytes, adolescents, immune regulation, concurrent training, systemic inflammatory response index

## Abstract

Physical exercise can have acute or short-term effects on immunological overnight recovery. Concurrent training (CT) is an often-applied exercise regime in team (e.g., soccer) and individual sports (e.g., judo, rowing) characterized by high training volumes and/or intensities. CT can be programmed in different sequencing schemes including strength/power before endurance training or *vice versa*. Here, we aimed to examine the acute effects (one exercise session) of different CT sequencing schemes on immunological recovery in young athletes. Male judo athletes (Tier 3, highly trained, national level) aged 16.0 ± 1.8 years were recruited to participate in a crossover repeated measures study design. Participants performed a power-endurance and an endurance-power CT sequence on separate days. Immunological stress regulation using capillary blood markers were tested immediately after the CT session and the night before (baseline) and after (intervention) CT sessions. Baseline evening measurements were performed from 5:00 p.m. to 7:00 p.m. and intervention evening measurements 6 h after the CT. Capillary blood markers were taken from the earlobe, plasma volume corrected, referenced to baseline and analyzed for order-by-time interactions using a generalized estimating equations statistical approach. White blood cells (p < 0.05), granulocytes (p < 0.001), the systemic inflammation index (p < 0.05), and the systemic inflammation response index (p < 0.001) showed significant group-by-time interactions. In contrast, monocytes, lymphocytes, and platelets did not exhibit a significant group-by-time effect (p > 0.05). Results were adjusted for repeated measurements using Bonferroni-Holm correction, which showed a significantly (p < 0.001) stronger immunological overnight regulation for granulocytes and the systemic inflammation response index following the power-endurance sequencing scheme, whereas white blood cells, lymphocytes, monocytes, blood platelets, and the systemic inflammation index did not show significant group-by-time interactions (p > 0.05). The observed findings for granulocytes and the systemic inflammation response index might be related to altered systemic stress regulation after the training session as the power-endurance sequence showed a higher increase in granulocytes on the evening after the exercise. Sleep behavior could affect the immunological systemic recovery and should therefore be further studied in future research. If immunological stress is already present, the endurance-power sequencing scheme appears to be more appropriate.

## 1 Introduction

Staying healthy is particularly important for athletes to be able to realize high training volumes and/or intensities for performance development. Long-term athlete development is a structured pathway including six stages with the goal to increase the likelihood to turn a talented young athlete into an elite athlete in a specific sport. Young athletes are confronted with many challenges on their developmental pathway to a high-performance athlete. Staying injury-free and healthy is an important prerequisite for successful athletic development (Bergeron et al., 2015). In team sports such as soccer or handball and in individual sports such as rowing or judo, adequate levels of strength, power and endurance are needed for athletic development (Franchini et al., 2011a). Accordingly, within a training day, strength and endurance training can be combined in different sequencing schemes, for instance on the level of an exercise session (e.g., strength training before endurance training or *vice versa*) (Coffey and Hawley, 2017). The combination of strength and endurance training has previously been described as concurrent training (CT). Irrespective of the CT programming approach, the order of strength and endurance training (sequencing) can moderate CT effects on different performance measures. If the goal is to maximize CT effects on physical performance, it is key for young athletes to stay healthy and avoid infections. While the long-term effects of physical exercise on the immune system have been well-documented (Gleeson et al., 2000; Moreira et al., 2013; Freitas et al., 2016; Moraes et al., 2017; Puta et al., 2018), the specific implications of short-term CT effects remain a dynamic area of research. Intense strength and endurance training have been shown to induce acute immunological stress responses in adolescent athletes, increasing capillary blood markers such as white blood cells (WBC), including granulocytes (GRAN), and lymphocytes (LYM) (Puta et al., 2018; Puta et al., 2018). Moreover, there is preliminary evidence that different CT sequencing schemes may impact on immunological outcomes (Mikkola et al., 2012; Cadore et al., 2014). Understanding how the CT sequencing scheme moderates immunological responses is crucial to specifically tailor CT regimes that have the potential to maximize both, performance and health in young athletes.

There is preliminary evidence showing that immunological stress responses appear to be affected by the applied CT sequencing schemes in young athletes (Markov et al., 2023). More specifically, the sequencing of power training before endurance training applied in one exercise session resulted in significantly greater pre-to-post exercise elevations in WBC and LYM immediately after the exercise session. In contrast, the endurance-power sequence produced notably larger pre-to-post increases in the GRAN-LYM ratio and the systemic inflammation index. Additionally, after 6 h of the applied CT session, higher pre-to-post increases were found for WBC and GRAN following power-endurance but not endurance-power training.

Different types of physical exercise applied at high intensities may have acute or short-term negative effects on immunological overnight recovery. Previous studies have investigated various factors influencing immunological regulation (Born et al., 1997; Kerkhofs et al., 2007; Faraut et al., 2011; Scheiermann et al., 2013; Pérez de Heredia et al., 2014; Irwin, 2019; Steidten et al., 2021; Wang et al., 2022; Ding et al., 2024). Immune cells are known to follow a circadian rhythm (Born et al., 1997; Wang et al., 2022; Ding et al., 2024). Lymphocyte levels peak in the evening and reach their lowest point in the morning, a pattern influenced by T-cell homing (Born et al., 1997; Wang et al., 2022). In contrast, neutrophil counts exhibit an inverse pattern, increasing during the night (Scheiermann et al., 2013). Sleep restriction has been shown to disrupt this circadian regulation of immune cell kinetics. (Kerkhofs et al., 2007). demonstrated that limiting sleep to only 4 h per night leads to a significant increase in WBC, neutrophil granulocytes, and monocyte concentrations, which may be attributed to the activation of the hypothalamus-pituitary-adrenal axis and the sympathetic nervous system (Irwin, 2019). Further evidence from (Faraut et al., 2011) suggests that recovery sleep, whether in the form of a power nap or a full 8-h sleep period, can restore leukocyte levels following sleep restriction; however, this effect was not observed after a standard 8-h recovery night alone. Additionally, (Steidten et al., 2021), reported that overnight immunological regulation following neuromuscular training is correlated with sleep duration in adolescent track and field athletes. While previous research has examined various factors influencing immunological regulation, the specific effects of CT sequencing on overnight immunological recovery remain largely unexplored in the literature.

Here, we aimed to explore the acute or short-term (one exercise session) effects of different CT sequencing schemes on overnight changes in capillary immunological blood markers in male young athletes.

With reference to previous studies from our laboratory (Steidten et al., 2021; Markov et al., 2023), we hypothesized that the overnight immune regulation in response to different CT sequencing schemes may be characterized by distinct changes in leukocytes and systemic inflammatory regulation. Specifically, we assume that the greater immunological stress induced by PE may trigger a stronger immune regulatory response overnight, potentially facilitating the return of WBC, GRAN, and SIRI to baseline levels and restoring homeostatic balance by the following morning.

## 2 Materials and methods

### 2.1 Study design

**FIGURE 1:**
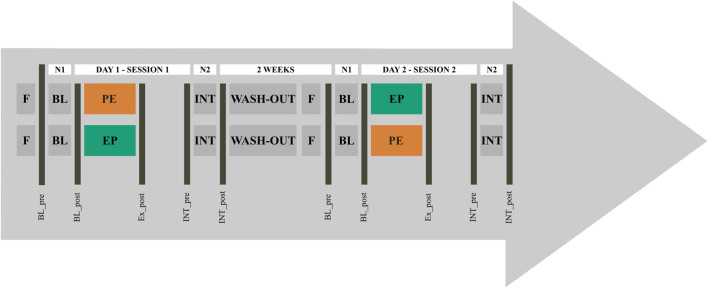
Study design for concurrent training sequencing for crossover repeated measures. The protocol started with a day without training (F). Concurrent training (CT) order was defined as power-endurance (PE) and endurance-power (EP). From each athlete, capillary blood markers were taken in the evening and morning before (BL_pre, BL_post) and after PE and EP (Ex_post, INT_pre, INT_post), reflecting night 1 (N1) and night 2 (N2).

This research project applied a crossover study design (see Figure 1). Participants were randomly assigned to two experimental groups. Before the start of the study, athletes from both groups were familiarized with CT procedures. While experimental group I performed power training before endurance training (PE) in one exercise session, group II followed the sequencing scheme endurance training before power training (EP). After a wash-out period of 2 weeks with regular and sport-specific exercise routines but without CT and a day off prior to session 2, group I followed the sequencing scheme of group II and *vice versa*. The training intensity was not quantified during wash-out phase. Over a period of four nights, athletes were monitored for changes in capillary immunological blood markers. For this purpose, blood samples were collected in the evening and morning before (BL; BL_pre, BL_post) and after (INT; INT_pre, INT_post) the intervention, as well as immediately following the CT exercise session (Ex_post). Because of the COVID-19 restrictions, participants had to arrive in three groups between 7:00 a.m. and 11:30 a.m. The BL_pre-measurements were scheduled between 5:00 p.m. and 7:00 p.m. and INT_pre 6 h after the training sessions.

### 2.2 Participants

Our participants were recruited from a youth academy judo performance center. Twenty male judo athletes aged 16.0 ± 1.8 years (body height: 171.2 ± 8.0 cm, body mass: 64.7 ± 11.6 kg) were recruited according to the conceptual model of long-term athlete development (as described by Granacher et al. (2016)). Participants’ chronological age ranged from 13 to 19 years. The maturity status was assessed using Tanner stages and all athletes were classified (Tanner stages 3–4). The training and performance calibre of the recruited athletes was highly-trained, national level (Tier 3) (McKay et al., 2022). Thus, the enrolled athletes regularly participated in national and/or international competitions. The participating athletes performed two daily training sessions on 5 days per week. Each training session lasted one to 2 h. Additionally, individuals with acute injuries or those reporting infectious diseases before or at any point during the experimental period were excluded from study participation. Due to the complexity of our statistical approach, statistical power was not conducted *a priori*. Participants and their legal representatives were informed about the experimental procedures, risks and benefits of the study. Written informed consent was obtained from the participating athletes and their legal representatives. This study was approved by the University Human Ethics Committee of the Potsdam University, Germany.

### 2.3 Exercise procedure

All athletes performed a warm-up routine with exercises from the FIFA 11+ program. Afterwards, CT was started in the group specific order. The muscle power exercise routine was performed on a leg-press machine (SCHNELL, Peutenhausen, Germany) with four sets of eight repetitions at 30%–40% of the individual’s 1-repetition maximum. Repetitions were carried out as fast (explosive) as possible. A 4 min rest between the sets was allowed. The exercise protocol is in line with the recommendations from the national strength and conditioning association (Bergeron et al., 2015). The Special-Judo-Fitness-Test (Sterkowicz et al., 1999) was used as sport-specific anaerobic endurance exercise. For this purpose, two opponents were placed 12 m apart and the athletes had to be thrown as often as possible with the ippon-seoinage technique (Franchini et al., 2011b). Ippon-seoinage is one of the many throwing techniques (nage-waza) in judo and the term can be translated to “shoulder throw.” Participants performed four rounds consisting of three sets with 15, 30, and 30 s duration with a break of 10 s between the sets and 4 min between the rounds. The total work time for each exercise was approximately 25 min.

### 2.4 Capillary immunological blood markers

WBC, GRAN, LYM, monocytes (MON), hemoglobin (HGB), hematocrit (HCT) and platelets (PLT) were measured using a 20 µL capillary blood sample taken from the earlobe. Analysis was performed using a medonic hematology system (Medonic M32, Boule Medical AB, Spånga, Sweden). Granulocytes are primarily involved in the acute inflammatory response and serve as the first line of defense against pathogens. Lymphocytes are central to the adaptive immune system, with T cells playing roles in cell-mediated immunity and B cells responsible for producing antibodies that target specific antigens. This specificity allows for a long-lasting immune memory against previously encountered pathogens. Monocytes, which can differentiate into macrophages and dendritic cells upon migration into tissues, are essential for phagocytosis, antigen presentation, and the regulation of immune responses. They act as a bridge between the innate and adaptive immune systems, helping to initiate and shape the immune response. Taking changes in plasma volume following exercise into account, capillary blood markers were corrected with respect to Dill and Costill (Dill and Costill, 1974) using the following formula:

$$Plasma\;volume\;change\;\left[\%\right]=100\;x\;\left[\frac{{HGB}_{pre}}{{HGB}_{post}}\;x\;\left(\frac{1-0,01*{HCT}_{post}}{1-0,01*\;{HCT}_{pre}}\right)\right]\;-100$$

$$Corrected\;values=not\;correted\;values\;x\;\left[\frac{100-Plasma\;volume\;change\;\left[\%\right]}{100}\right]$$

Corrected values were used to calculate the systemic inflammation index (SII). Therefore, the following formula (Hu et al., 2014) was used:

$$SII=PLT*\frac{GRAN}{LYM}$$

Furthermore, the systemic inflammatory response index (SIRI) was calculated with reference to (Yi et al., 2021; Bai et al., 2024):

$$SIRI=\frac{GRAN*MON}{LYM}$$

### 2.5 Statistics

**FIGURE 2:**
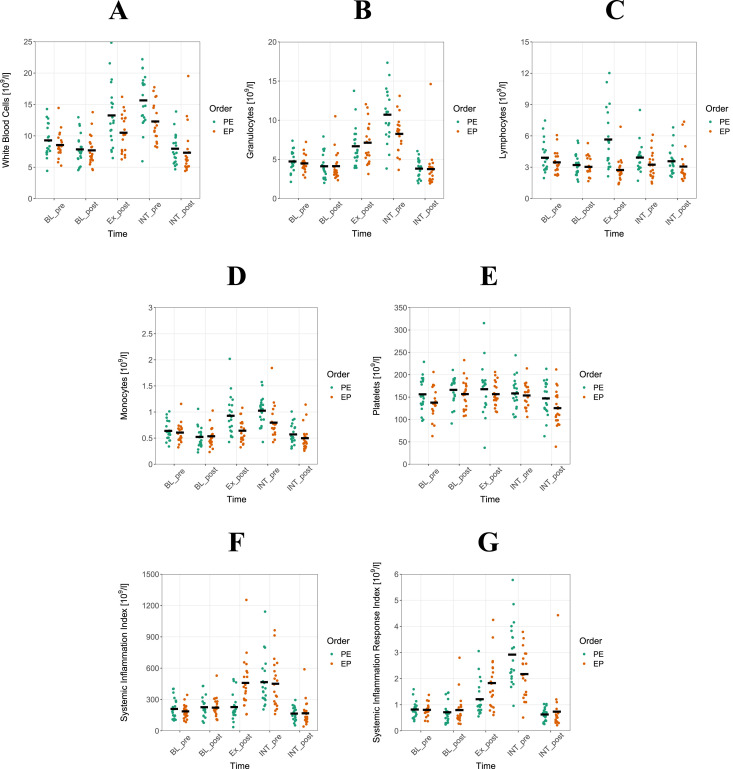
Granulocytes have impact for systemic inflammation response index in Concurrent training. Shown are means of white blood cells (**(A)**, single data), granulocytes **(B)**, lymphocytes **(C)**, monocytes **(D)**, platelets **(E)**, systemic inflammation index **(F)** and systemic inflammation response index **(G)** for power-endurance (PE) and endurance-power (EP) concurrent training (CT). From each athlete, measurements were taken in the evening and morning before (BL_pre, BL_post) and after PE and EP (INT_pre, INT_post) as described in Figure 1. Altogether, measurements of 20 athletes are displayed.

The raw data is included in Supplementary Material 1 and plasma-volume corrected data are presented in Figure 2. The descriptive statistics can be found in Supplementary Material 2. To account for repeated measurements and randomly missing data, order (PE, EP) ^*^ time (BL, INT) interactions were analyzed using a generalized estimating equation (GEE) approach. GEE is known to produce robust parONameter estimates and standard errors (Zeger and Liang, 1986; Burton et al., 1998). Plasma volume-corrected evening to morning change-scores for BL and INT were reported as marginal means and 95% confidence intervals. BL was used as baseline. Change-scores of INT were referenced to BL change scores using z-transformation. Capillary blood markers were entered separately as dependent variables. Order, time and order ^*^ time were used as independent variables. Within subjects, dependencies were modeled as first-order autoregressive and a Gaussian link function was used. With reference to Fritz et al. (2012), effect sizes were classified as large, medium and small effects (0.5; 0.3; 0.1). The significance level was set to α = 5%. To account for multiple comparison, we reported Bonferroni-Holm corrected p-values. Data analyses was performed using R version 4.2.2 (R Foundation for Statistical Computing, Vienna, Austria) and “geepack” package (Yan, 2002; Yan and Fine, 2004; Halekoh et al., 2006).

## 3 Results

Marginal means and 95% confidence intervals for plasma volume corrected and referenced evening to morning change-scores for BL and INT and their differences are displayed in Table 1. After Bonferroni Holm correction, the results demonstrated that following the PE CT sequence, capillary blood values for WBC (βz = −2,860, 95% CI: −3.61 to −2.11, p < 0.001), GRAN (βz = −4.980, 95% CI: −6.02 to −3.94, p < 0.001), MON (βz = −1.760, 95% CI: 2.46- to −1.06, p < 0.001), SII (βz = −4.270, 95% CI: −5.68 to −2.86, p < 0.001) and SIRI (βz = −6.980, 95% CI: −8.58 to −5.38, p < 0.001) exhibited a significantly greater reduction during INT (evening-to-morning change after the intervention) compared to BL (evening-to-morning change before the intervention). Furthermore, PLT (βz = −0.966, 95% CI: −1.68 to −0.26, p < 0.05) showed a significant reduction. Following the EP CT sequence, GRAN (βz = −2.070, 95% CI: −3.10 to −1.04, p = 0.001), SII (βz = −2.410, 95% CI: −3.38 to −1.44, p < 0.001) and SIRI (βz = −2.410, 95% CI: −3.38 to −1.44, p = 0.001) exhibited a significantly greater reduction during INT. Similarly, WBC (βz = −1.540, 95% CI: −2.44 to −0.64, p < 0.05) and PLT (βz = −1.030, 95% CI: −1.60 to −0.46, p < 0.05) also showed a significantly greater reduction during this period. Regarding MON, it is noteworthy that a significantly greater reduction during INT was observed only under the PE condition. Under the EP condition, the significance threshold was narrowly missed (p = 0.057). For LYM, no significant differences were found between BL and INT following either the PE or EP scheme.

**TABLE 1:** Results for the night before (BL) and after (INT) Concurrent training (CT) with differences.

Measure	Unit	Order	BL			INT			Difference BL - INT					
			Mean	95% CI		Mean	95% CI		βz	SE	95% CI		p	p_adj_
WBC	z-score	PE	0.00	−1	1	−2.86	−3.47	−2.25	−2.860	0.383	−3.61	−2.11	0.000	**0.000**
	z-score	EP	0.00	−1	1	−1.54	−2.33	−0.75	−1.540	0.460	−2.44	−0.64	0.001	**0.004**
GRAN	z-score	PE	0.00	−1	1	−4.98	−5.93	−4.03	−4.980	0.533	−6.02	−3.94	0.000	**0.000**
	z-score	EP	0.00	−1	1	−2.07	−3.00	−1.14	−2.070	0.525	−3.10	−1.04	0.000	**0.001**
LYM	z-score	PE	0.00	−1	1	0.28	−0.39	0.96	0.283	0.409	−0.52	1.08	0.490	0.490
	z-score	EP	0.00	−1	1	0.26	−0.23	0.75	0.261	0.336	−0.40	0.92	0.440	0.880
MON	z-score	PE	0.00	−1	1	−1.76	−2.30	−1.22	−1.760	0.355	−2.46	−1.06	0.000	**0.000**
	z-score	EP	0.00	−1	1	−1.02	−1.75	−0.29	−1.020	0.435	−1.87	−0.17	0.019	0.057
PLT	z-score	PE	0.00	−1	1	−0.97	−1.52	−0.41	−0.966	0.362	−1.68	−0.26	0.008	**0.030**
	z-score	EP	0.00	−1	1	−1.03	−1.40	−0.66	−1.030	0.293	−1.60	−0.46	0.000	**0.003**
SII	z-score	PE	0.00	−1	1	−4.27	−5.61	−2.93	−4.270	0.717	−5.68	−2.86	0.000	**0.000**
	z-score	EP	0.00	−1	1	−2.41	−3.27	−1.55	−2.410	0.493	−3.38	−1.44	0.000	**0.000**
SIRI	z-score	PE	0.00	−1	1	−6.98	−8.52	−5.44	−6.980	0.82	−8.58	−5.38	0.000	**0.000**
	z-score	EP	0.00	−1	1	−2.31	−3.37	−1.25	−2.310	0.58	−3.45	−1.17	0.000	**0.001**

Marginal means and confidence intervals (95% CI) resulting from the Generalized Estimating Equation models for the plasma volume corrected and referenced change-scores of capillary blood markers. Blood markers were referenced to the night before the concurrent training (CT). Order specific (PE, CT power-endurance order; EP, CT endurance-power order) differences between BL and INT were evaluated and are presented as p-values (p) und Bonferroni Holm corrected p-values (p_adj_).

**FIGURE 3:**
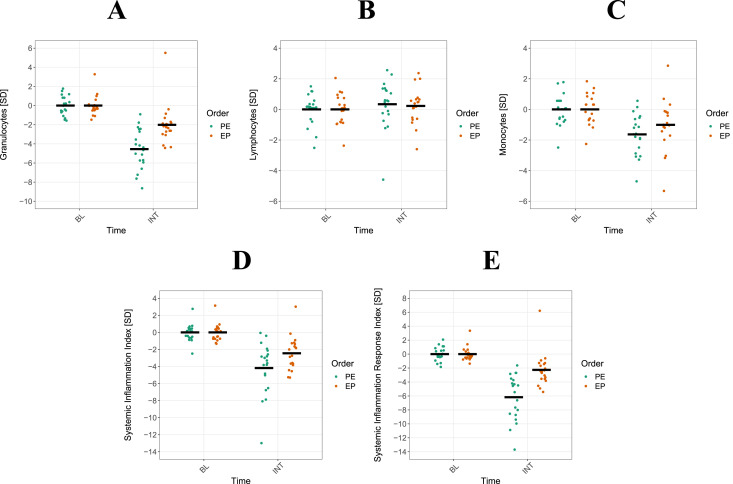
Interaction between power-endurance (PE) and endurance-power (EP) concurrent training (CT). Shown are means and single data for 20 athletes. Sequencing interactions of PE and EP between the night before (BL) and after (INT) CT are displayed for granulocytes (**(A)**, single data), lymphocytes **(B)**, monocytes **(C)** systemic inflammation index **(D)** and systemic inflammation response index **(E)**.

The results of the univariate regression analyses using GEE models are displayed in Table 2 and Figure 3. The analysis identified significant order-by-time interactions, which demonstrated a notably greater reduction in WBC (βz = 1.322, 95% CI: 0.22 to 2.50, p < 0.05), GRAN (βz = 2.910, 95% CI: 1.44 to 4.38, p < 0.001), SII (βz = −1.860, 95% CI: −0.15 to −3.57, p < 0.05) and SIRI (βz = 1.860, 95% CI: 2.71 to 6.63, p < 0.001) for the PE CT sequence compared to EP. After adjusting for repeated measurements, only GRAN and SIRI remained significant (p < 0.001). Although WBC, MON, PLT, and SII exhibited a significantly greater reduction during INT (evening-to-morning change after the intervention) in both CT sequencing schemes, no significant order-by-time interaction was observed (p < 0.05). A similar result was observed for LYM, which do not appear to be influenced by the different CT schemes.

**TABLE 2:** Order-by-time interactions.

Measure	Unit	Order*Time					
		βz	SE	95% CI		p	p_adj_
WBC	z-score	1.322	0.599	0.15	2.50	**0.027**	0.135
GRAN	z-score	2.910	0.748	1.44	4.38	**0.000**	**0.001**
LYM	z-score	−0.021	0.529	−1.06	1.02	0.970	0.970
MON	z-score	0.740	0.562	−0.36	1.84	0.190	0.570
PLT	z-score	−0.064	0.465	−0.98	0.85	0.891	0.999
SII	z-score	1.860	0.871	−3.57	−0.15	**0.033**	0.132
SIRI	z-score	4.670	1.000	2.71	6.63	**0.000**	**0.000**

Results from the Generalized Estimating Equation models for order*time interactions are presented as p-values (p) und Bonferroni Holm corrected p-values (p_adj_).

## 4 Discussion

### 4.1 Impact of exercise sequencing on immunological recovery

The present study explored the sequencing effects of CT either applied as PE or EP on immunological recovery in young male judo athletes. The key findings revealed significant order-by-time interactions for WBC, GRAN, SII and SIRI. However, after Bonferroni Holm correction, only GRAN and SIRI remained significantly affected. These results suggest that the sequence of power and endurance exercises within CT plays a crucial role in influencing immunological regulation overnight.

Strength training is known to result in an increased release of interleukin-6 (IL-6) from muscle fibers, which facilitates immunological regulation for the repair of damaged muscle tissue through satellite cells (Izquierdo et al., 2009; Ihalainen et al., 2017). The increase in IL-6 is dependent on the type of mechanical load. Ihalainen et al. (2014) demonstrated that an accentuated eccentric load, as in hypertrophy training, leads to a greater release of IL-6 and cortisol compared to maximal strength training within a 30 min period post training. Intense endurance training induces an increase in cortisol levels, which rise several hours post-exercise, causing a delayed immunological stress response, characterized by lymphopenia and neutrophilia (Gabriel and Kindermann, 1997). Although the total load is the same in both CT sequencing schemes, significant differences in overnight immunological regulation are evident. The PE sequence resulted in a stronger immunological response overnight. This varying response is likely due to the restoration of immunological homeostasis during the night. The underlying mechanism for the different stress responses during the day and the resulting overnight regulation remains unknown. Due to the different immunological effects of EP and PE within CT, EP appears to cause a smaller disruption of immunological homeostasis compared to PE. CT order influences immunological systemic recovery overnight, which highlights its importance for health prevention. Therefore, EP should be preferred, especially in the presence of mild upper respiratory symptoms.

CT and the sequencing of CT influenced myeloid cells (GRAN and MON) but had no effect on lymphoid cells (LYM). This suggests that CT impacts the immunological regulation of the innate immune system but not the adaptive immune system. GRAN play a crucial role in countering bacterial infections. Although GRAN, such as neutrophil granulocytes, operate through phagocytosis, they are not classified as antigen-presenting cells (APCs), which bridge the innate and adaptive immune systems by facilitating T-cell activation. Monocytes, in contrast, are highly dynamic and play a dual role in immune regulation and tissue repair. Exercise-induced mobilization of monocytes often results in their infiltration into skeletal muscle, where they differentiate into tissue-resident macrophages that support repair and regeneration, particularly after intense exercise causing muscle damage (Peake et al., 2017). Monocytes also differentiate into dendritic cells, which, along with macrophages, are among the most important antigen-presenting cells (APCs) responsible for activating the adaptive immune system. In our study, the observed effect of CT on GRAN but not MON suggests that CT primarily disrupts immunological homeostasis within the innate immune system, without significantly affecting the complex, context-dependent roles of monocytes in inflammation and tissue repair.

The observed order-by-time interaction in GRAN may be linked to altered endocrine responses after the training session. Notably, the power-endurance CT showed a higher increase in GRAN on the evening after the exercise compared to endurance-power CT. This differential response might be indicative of varying physiological stressors imposed by different exercise sequences, impacting the immune system differently (Bessa et al., 2016). Furthermore, the CT order affects immunological systemic recovery overnight, which highlights its importance for health prevention.

These findings align with prior work of (Cadore et al., 2014) who delved into the immune responses to CT. The study supports the notion that understanding the order of power and endurance exercises is paramount for optimizing the immune response in athletes. The present study builds upon this foundation by specifically focusing on young male athletes and emphasizing the importance of immunological recovery overnight.

An examination of the raw data in Figure 2 reveals that values at the INT_post time point return to levels observed at BL_post. While this may suggest that the CT sequencing effect has only a short-term impact, considering the findings of Steidten et al. (2021), it is expected that differential overnight immunological regulation could result in a reduction after three consecutive days of CT. This reduction, likely influenced by varying levels of immune system strain, may also exhibit an interaction effect. However, based on the present data, this remains speculative.

### 4.2 Sleep behavior and caloric intake as a moderator

Considering the intricate relationship between sleep and immune function, the observed changes in immunological markers may also be influenced by sleep behavior. Sleep quality and duration are known to impact immune parameters (Prather et al., 2015; Besedovsky et al., 2019). Therefore, quantitative variations in immunological markers observed in the study could be attributed, at least in part, to differences in sleep patterns among participants.

This aligns with the sleep-immune interactions highlighted by Steidten et al. (2021). The study emphasizes the need for considering sleep behavior in investigations related to immune responses in athletes. Future research should delve deeper into the interplay between exercise sequencing, sleep, and immunological recovery, as understanding these multifaceted relationships is crucial for optimizing training protocols.

In addition to sleep, recent research has highlighted the impact of dietary intake and meal timing on our immune system. Jordan et al. (2019) have indicated that fasting periods, specifically those lasting around 16 h, can prompt monocytes to migrate to the bone marrow, where they undergo regeneration. This finding suggests a link between dietary habits and immune cell dynamics and the need for standardized nutritional patterns. However, in this study, caloric intake and meal timing were not standardized due to the challenges posed by the COVID-19 pandemic and the associated individual measurements. Consequently, the results of this study may be influenced by the variations in meal times and caloric intake among participants.

### 4.3 Implications for training strategies

The implications of these findings for training strategies applied in young male judo athletes are substantial. Given the observed differences in immunological responses based on the order of power and endurance exercises, coaches and strength and conditioning specialists should tailor training programs to prioritize immunological recovery. Specifically, when immunological stress is already present, the study suggests that the EP sequencing scheme may be the preferred option, as it results in less immune system disruption compared to the PE sequencing scheme. However, for healthy athletes without signs of immune stress or upper respiratory symptoms, both sequencing options (EP and PE) allow for effective immunological recovery overnight, returning to baseline levels by the morning. This means that, under normal circumstances, healthy athletes are unlikely to experience significant immune differences the following day. On the other hand, when signs of mild upper respiratory symptoms, such as a sore throat or runny nose, are present, the study recommends choosing the endurance-first sequencing scheme (EP) to minimize the immunological burden and ensure better recovery. This consideration is crucial in optimizing training and recovery strategies to support long-term athlete health and performance.

Moreover, incorporating interventions to enhance sleep quality and duration may further contribute to optimal immunological recovery in young athletes. This aligns with the broader context of ensuring the holistic development of young sports talents, as emphasized in the introduction.

### 4.4 Limitations and future directions

Despite the insights provided by this study, it is essential to acknowledge its limitations. The small sample size and the focus on male judo athletes from a national training center may limit the generalizability of the findings. Previous research indicates that women typically perform more repetitions at submaximal loads, such as 80% of 1-RM, compared to men. This is largely due to gender-specific differences in muscle fatigue resistance and fiber composition, with women often exhibiting a greater proportion of Type I muscle fibers, which are more resistant to fatigue (Falch et al., 2023). To ensure more consistent and interpretable results, we focused on male athletes. However, similar results are expected for females.

A classic cross-over design was not feasible in our study due to practical limitations, meaning that we could not fully assess intersubject variability as no participant could complete both sequences (A,B and B,A; respectively EP-PE and PE-EP). Consequently, we opted for a between-subject design. Given these conditions, it was predetermined that a pooled analysis using a GEE model represented one of the most appropriate methodological choices. Statistical power is influenced by both sample size and the number of repeated measures. However, our results can only be interpreted in the context of the overall group of participants. An interpretation regarding intrasubject effects is not possible based on our analysis.

He Generalized Estimating Equations (GEE) method does not require a specific distribution of outcome measures. GEE is suitable for small sample sizes (n = 8) and repeated measures (n = 6), achieving approximately 80% power (Ma et al., 2012). A *post hoc* power analysis was performed for WBC with an order × time interaction effect of 0.92 and a Type I error rate of 0.05. The results indicated a statistical power of 100%.

Future research should aim for larger and more diverse samples to strengthen the external validity of the results.

Additionally, further investigations should explore the underlying mechanisms of altered immune responses and regulation overnight. Longitudinal studies tracking sleep patterns, hormonal fluctuations, and immune responses over an extended period would offer a more comprehensive understanding of these complex interactions.

## 5 Conclusion

This study contributes valuable insights into the intricate interplay between exercise sequencing with CT, sleep, and immunological recovery in young male judo athletes. Specifically, a single CT session following the power-endurance (PE) order results in stronger overnight immunological regulation, allowing both granulocytes and the systemic inflammation response index to return to baseline levels. However, given that the effects under conditions of immunological stress, such as a mild upper respiratory infection, have not yet been investigated, it is recommended to prioritize the endurance-power (EP) sequencing in such circumstances to ensure a more favorable immune response.

## Data Availability

The original contributions presented in the study are included in the article/Supplementary Material, further inquiries can be directed to the corresponding authors.
